# Editorial: Catalytic production of sustainable fuels and derivatives via carbon footprints

**DOI:** 10.3389/fchem.2024.1465517

**Published:** 2024-08-14

**Authors:** Muhammad Asif Nawaz, José Antonio Odriozola, Jie Yu

**Affiliations:** ^1^ Department of Inorganic Chemistry, University of Seville, Seville, Spain; ^2^ Huazhong University of Science and Technology, Wuhan, China

**Keywords:** sustainable energy (SE), biofuels production, catalytic materials for energy, reaction engineering (REA) approach, CO_2_ valorisation

## 1 Introduction

The urgency of combating climate change requires a transition to sustainable energy systems, with advanced catalytic processes playing a crucial role (Blay-Roger et al.). However, this transition faces significant challenges, including the entrenched reliance on fossil fuels and the need to overcome technical, economic, and infrastructural barriers (Blay-Roger et al., 2024b). One of the foremost challenges is the entrenched reliance on fossil fuels, which are deeply embedded in our industrial and economic systems Where, shifting to renewable resources such as biomass and CO_2_ requires overcoming significant technical, economic, and infrastructural barriers (Nawaz et al., 2023). Technically, developing efficient and selective catalysts that operate under mild conditions is essential to maximize product yield and minimize waste, while also addressing catalyst stability and resistance to deactivation (Fanhui et al., 2022). Economically, substantial initial investments and comprehensive life cycle assessments are needed to ensure the viability of new catalytic processes (Blay-Roger et al., 2024a). Logistically, integrating these processes into existing industrial frameworks requires strategic planning and policy support. Infrastructurally, transitioning involves significant changes to the energy grid and supply chains, necessitating reliable renewable feedstocks and efficient conversion methods. Interdisciplinary collaboration is vital for addressing these complex challenges. The Research Topic “Catalytic Production of Sustainable Fuels and Derivatives via Carbon Footprints” highlights advancements in catalytic technologies that reduce carbon emissions and enhance environmental sustainability. Catalysis, historically central to the chemical industry, is evolving to meet sustainability principles by converting renewable resources into valuable products. This Research Topic addresses key challenges and strategies for improving catalytic efficiency and selectivity, contributing to sustainable and economically viable processes. It underscores the importance of advanced material science and chemical engineering in fostering sustainable energy practices, providing both scientific understanding and practical solutions for real-world applications, paving the way for a greener future.

## 2 Theme and significance

One of the critical questions addressed in this Research Topic is how to improve the efficiency and selectivity of catalysts to maximize the yield of desired products while minimizing by-products and waste. This involves optimizing catalyst composition, structure, and reaction conditions. Another significant Research Topic is the utilization of sustainable feedstocks, such as biomass and CO_2_, for fuel and chemical production. Research in this Research Topic explores how advanced catalysts can facilitate the efficient conversion of these renewable resources, contributing to a circular economy and reducing carbon emissions. Integrating advanced catalytic processes into existing industrial frameworks poses both opportunities and challenges. Therefore, discussing strategies for scaling up laboratory-scale innovations to industrial applications and addressing economic and logistical considerations is essential. Understanding and mitigating the environmental impact of catalytic processes is paramount. This includes evaluating the life cycle of catalysts, their recyclability, and the overall carbon footprint of the processes they enable. Additionally, the stability and deactivation of catalysts are critical concerns that require innovative solutions to extend catalyst lifetimes and enhance their performance. The complexity of developing sustainable catalytic processes necessitates interdisciplinary collaboration. This Research Topic highlights the integration of insights from material science, chemical engineering, environmental science, and economics to create holistic solutions. Addressing the social and ethical implications of these technologies, such as ensuring equitable access to sustainable energy and minimizing negative impacts on communities, is also crucial for fostering a just energy transition.

## 3 Articles

### 3.1 CO_2_ hydrogenation to methanol: catalyst optimization

The study by Liu et al., examines the impact of the Mg/Al molar ratio on CuMgAl-x catalysts for CO_2_ hydrogenation to methanol. The right Mg/Al ratio improves Cu dispersion and CO_2_ adsorption, resulting in higher methanol selectivity and conversion rates. This research provides valuable insights for catalyst design and industrial CO_2_ utilization, contributing to carbon capture and the production of valuable chemicals and fuels.

### 3.2 Enhancing light olefins selectivity via catalytic cracking

Ali et al., explore the catalytic cracking of n-hexane using ZSM-5 catalysts modified with lanthanum and phosphorus. This modification improves the catalyst’s acidity and stability, increasing the yield of light olefins. The study enhances the understanding of catalyst design and has practical implications for optimizing olefin production in the petrochemical industry, aiming for more efficient and environmentally friendly processes.

### 3.3 Innovations in Pt-based electrocatalysts for carbohydrate oxidation

In the third article, Castañeda-Morales et al., investigate Pt-based electrocatalysts on carbon supports for carbohydrate electro-oxidation. The study highlights the importance of support materials, with carbon Vulcan and carbon black showing different behaviors in oxidizing glucose, fructose, and sucrose. Insights into the structural, morphological, and electronic characteristics of these catalysts are crucial for designing efficient and selective electrocatalysts, important for direct fuel cells and biofuel production.

### 3.4 Bioenergy innovations for a sustainable future

Blay-Roger et al., review bioenergy technologies, focusing on biofuels like renewable diesel, bio jet fuel, and ethanol for reducing greenhouse gas emissions. The article discusses technological advancements, policy frameworks, and the need for cross-sector collaborations to address challenges like feedstock supply consistency and decentralized processing units. It advocates for a systemic shift towards sustainable energy practices, emphasizing the comprehensive nature of bioenergy solutions.

## 4 Conclusion and future directions

The research in this Research Topic advances catalysis and supports sustainable development goals by reducing the carbon footprint of fuel and chemical production. It highlights significant steps towards a sustainable and environmentally friendly future, demonstrating critical advancements in catalysis and bioenergy. Where, future research in this field can focus on the following key areas:

• Developing catalysts with improved stability and resistance to deactivation for long-term industrial use through novel materials and surface modifications.
• Achieving breakthroughs in catalyst efficiency and selectivity by optimizing composition, structure, and reaction conditions to maximize desired product yields and minimize waste.
• Integrating advanced catalytic processes with renewable energy sources like solar and wind power to enhance system efficiency, reduce fossil fuel dependence, and lower carbon emissions.
• Conducting comprehensive life cycle assessments and economic viability studies to ensure environmental sustainability and commercial feasibility, including evaluating catalyst recyclability and overall process carbon footprint.
• Implementing robust policies and strategic investments to support scaling up innovations from the lab to industrial applications, leveraging government incentives, industry partnerships, and public-private collaborations.
• Ensuring equitable access to sustainable energy and minimizing negative impacts on communities by considering social and ethical implications in research.
